# National Assessment of Emergency, Intensive Care, and Anesthesia Physician Distribution in Saudi Arabia, 2023: Implications for Emergency Care Access

**DOI:** 10.1155/emmi/9606167

**Published:** 2026-04-11

**Authors:** Waleed M. Kattan

**Affiliations:** ^1^ Department of Health Services and Hospitals Administration, Faculty of Economics and Administration, King Abdulaziz University, Jeddah, Saudi Arabia, kau.edu.sa

**Keywords:** anesthesiologists, emergency medicine physicians, geographic inequality, health workforce distribution, intensive care physicians, physician density, Saudization

## Abstract

**Background:**

In line with Saudi Arabia’s Vision 2030, transforming the health workforce has become a central priority, particularly to address persistent geographic and sectoral inequities and imbalances in national workforce participation within critical specialties such as emergency medicine, intensive care, and anesthesia. Despite national workforce expansion, no comprehensive assessment has quantified the distribution of these physicians across regions and sectors.

**Objectives:**

This study aims to examine the distribution and density of emergency, intensive care, and anesthesia physicians across Saudi Arabia’s 20 health regions, assess disparities by sector and nationality, and quantify inequality using Gini coefficients.

**Methods:**

This cross‐sectional study utilized secondary data sourced from the Ministry of Health’s 2023 Statistical Yearbook. Physician counts were categorized by sector: the Ministry of Health, other governmental entities, and the private sector. Regional physician‐to‐population ratios were calculated, and inequality was assessed using Lorenz curves and the Gini coefficient.

**Results:**

In 2023, Saudi Arabia had 4999 emergency physicians, 4559 anesthesiologists, and 3185 intensivists, with approximately 65% employed by the Ministry of Health. Saudi workforce participation varied substantially, reaching 46% in emergency medicine but remaining lower in intensive care (37%) and anesthesia (22%). From 2019 to 2023, physician densities increased across all three specialties to 14.8 per 100,000 for emergency medicine, 13.5 for anesthesia, and 9.4 for intensive care, levels favorable relative to international benchmarks. Despite this growth, marked geographic disparities persisted: Riyadh and Jeddah had the largest absolute numbers but lower population‐adjusted densities, whereas smaller regions showed higher per‐capita ratios despite limited infrastructure. Geographic inequality was moderate across specialties, with Gini coefficients of 0.52 for anesthesia, 0.49 for intensive care, and 0.45 for emergency medicine.

**Conclusion:**

Despite overall workforce growth, Saudi Arabia’s acute care physicians remain unevenly distributed: urban centers house most staff but have lower per‐capita coverage, whereas peripheral regions have higher ratios but limited capacity. Addressing national workforce participation and optimizing regional deployment are essential for equitable care.

## 1. Introduction

Saudi Arabia is undergoing a significant transformation in its healthcare system, guided by Vision 2030 and the National Transformation Program (NTP) 2020 [1]. These strategic initiatives aim to improve access to services, enhance system sustainability, and reduce dependency on foreign labor by prioritizing Saudization efforts [2, 3]. This shift is particularly timely, given the mounting demand for healthcare driven by demographic trends, such as an aging population and an increasing burden of chronic diseases [4–6]. Despite these priorities, progress in national workforce participation (Saudization) [7] has been uneven across medical specialties, particularly in high‐acuity care.

Emergency medicine, anesthesia, and intensive care represent the cornerstone of acute care delivery in modern healthcare systems. Intensive care units provide specialized medical and nursing care with enhanced monitoring capacity, and multiple modalities of physiologic organ support to sustain life during periods of life‐threatening organ‐system insufficiency [8]. These specialties form an integrated continuum in which early, evidence‐based interventions can attenuate organ dysfunction and improve survival outcomes [9, 10]. Adequate emergency physician staffing and optimal geographic distribution are particularly critical, as workforce shortages and maldistribution can exacerbate ED crowding, prolong wait times, and compromise timely access to emergency interventions. Workforce shortages and geographic maldistribution can compromise the quality and responsiveness of emergency care, with peripheral areas often relying on general practitioners rather than board‐certified emergency specialists [11, 12]. At the national level, Saudi Arabia reports physician densities that exceed commonly cited international reference levels, with 14.8 emergency physicians, 13.5 anesthesiologists, and 9.4 intensivists per 100,000 population in 2023 (Table 1), compared with global benchmarks that typically range below 5 per 100,000 for emergency medicine [13] and anesthesia [14] and 2–5 per 100,000 for intensive care [15]. Despite this quantitative adequacy, geographic maldistribution and sectoral concentration remain important challenges to equitable access, healthcare system resilience, and emergency response capacity. These comparisons suggest that Saudi Arabia’s principal challenge lies not in absolute workforce scarcity but in the geographic distribution of physicians relative to population size and service capacity. However, the extent and pattern of this maldistribution have not been systematically quantified across all regions and sectors.

**TABLE 1:** Trends in the physicians workforce and population ratios in Saudi Arabia (2019–2023)^∗^.

Year	Population	Total emergency physicians (per 100,000 residents)	Total intensivists (per 100,000 residents)	Total anesthesiologists (per 100,000 residents)
2019	30,063,799	4077 (13.6)	2552 (8.5)	3661 (12.2)
2020	31,552,510	4291 (13.6)	2645 (8.4)	3775 (12.0)
2021	30,784,383	4422 (14.4)	2794 (9.1)	3836 (12.5)
2022	32,175,224	4699 (14.6)	2814 (8.7)	4165 (12.9)
2023	33,764,022	4999 (14.8)	3185 (9.4)	4559 (13.5)

^∗^Includes MOH, PS, and other governmental sectors.

Despite Vision 2030’s emphasis on workforce transformation and equitable access to healthcare, critical gaps in understanding workforce distribution persist [16, 17]. Although reform efforts have successfully expanded the overall physician workforce and achieved favorable national densities, the geographic distribution and sectoral balance of acute care specialists remain poorly characterized, particularly with respect to the alignment between physician supply and regional service capacity [4, 18]. Patients in many regions experience prolonged emergency department waiting times [11], and peripheral areas often rely on general practitioners rather than certified emergency specialists [12]. Furthermore, challenges in training pipelines, retention strategies, and career preferences, particularly the documented preference of anesthesia residents for international careers over domestic academic or public‐sector roles [19], threaten the sustainability of localization efforts. While these concerns have been documented in fragmented reports, no comprehensive national assessment has systematically quantified the distribution, density, and inequality of emergency medicine, anesthesia, and intensivists across Saudi Arabia’s health regions [20–23].

This study addresses a critical knowledge gap by examining the distribution and density of emergency medicine, intensive care, and anesthesia physicians across Saudi Arabia’s 20 health regions. Drawing on data from the Ministry of Health (MOH) Statistical Yearbook 2023 [24], the analysis characterizes sectoral composition (MOH, other governmental and private sectors [PS]), Saudization rates, and regional disparities. Geographic inequality is quantified using Lorenz curves and Gini coefficients. By providing the first comprehensive national assessment of these acute care specialties, the findings aim to inform data‐driven policy interventions that can advance healthcare equity, strengthen workforce planning, and optimize resource allocation in alignment with Vision 2030 objectives.

## 2. Methods

### 2.1. Study Design and Data Source

This cross‐sectional study analyzed the geographic and sectoral distribution of emergency medicine, intensive care, and anesthesia physicians in Saudi Arabia using secondary data from the MOH Statistical Yearbook 2023 [24]. The MOH Statistical Yearbook is an open, publicly available dataset published annually by the MOH for public use and research purposes, accessible without registration or institutional approval. No formal permission was required to access this publicly available dataset.

A census‐based sampling approach was used to include all practicing emergency physicians, intensivists, and anesthesiologists registered in the national health system in 2023. The MOH Statistical Yearbook classifies physicians within each specialty into three hierarchical training levels: residents, registrars, and consultants. For this study, all three training levels were combined within each specialty to provide comprehensive workforce totals for emergency medicine, intensive care, and anesthesia.

Data were obtained for each of the 20 health regions, including physician counts categorized by healthcare sector (MOH, other governmental sectors, and PS), corresponding population figures to calculate physician‐to‐population ratios, and hospital bed capacity (MOH and PS combined) to calculate physician‐to‐bed ratios as a complementary capacity‐based metric. Gender and nationality distributions were also obtained for analysis of workforce composition. Additionally, historical national workforce data for the period 2019–2023 were obtained to assess temporal trends in physician counts and population‐adjusted densities across the three specialties.

### 2.2. Workforce Density Measurement

Workforce density was measured using two complementary metrics: (1) the number of physicians per 100,000 residents and (2) the number of physicians per 100 hospital beds. The physician‐to‐population ratio per 100,000 residents was used as a standardized epidemiological metric to enable consistent comparison of workforce density across regions and specialties and facilitate international benchmarking. While anesthesia and intensive care medicine are predominantly hospital‐based specialties with service demand more closely linked to surgical volume and ICU bed capacity than to population size alone, using a common population‐based denominator facilitates comparability with national and international workforce studies and allows assessment of geographic equity. To address this limitation, physician‐to‐bed ratios (per 100 beds) were calculated as a secondary analysis to examine workforce distribution relative to hospital infrastructure capacity. This capacity‐based metric provides complementary insight into workforce adequacy relative to service‐delivery infrastructure, particularly in hospital‐based specialties. Specialty‐specific workload indicators, such as surgical volumes, ICU occupancy rates, and emergency department visits, were not uniformly available at the regional level; therefore, population‐adjusted density and bed‐based ratios were selected as the most feasible and transparent measures for cross‐specialty and cross‐regional analyses. Both metrics have been widely adopted in prior national workforce assessments, particularly when integrated demand‐side indicators are unavailable [17, 21, 25].

### 2.3. Inequality Analysis

To quantify regional inequality in physician distribution, Lorenz curves were constructed and Gini coefficients calculated for emergency physicians, intensivists, and anesthesiologists across all sectors. The analysis employed non‐population‐weighted data, treating each of Saudi Arabia’s 20 health regions as an independent geographic unit regardless of population size. For Lorenz curve construction, regions were ranked in ascending order by absolute physician count, and cumulative proportions were computed for both regions (*x*‐axis) and physicians (*y*‐axis) to plot against the 45‐degree line of perfect equality. The Gini coefficient was derived using the trapezoidal approximation formula:

(1) Gini=1−∑Xi+1−XiYi+1+Yi,

where *X*_*i*_ denotes the cumulative proportion of regions and *Y*_*i*_ denotes the cumulative proportion of physicians. Values range from 0 (perfect equality) to 1 (maximal inequality). Sector‐specific Lorenz curves and Gini coefficients were computed separately for MOH and PS distributions. All statistical analyses were conducted using R version 4.4.3 (GUI 1.81 Big Sur ARM build 8497).

### 2.4. Data Management

Physician data from other governmental sectors, such as military or National Guard institutions, lacked geographic attribution in the MOH Statistical Yearbook and were therefore excluded from regional analyses but included in overall national totals. This exclusion limits the ability to assess the complete regional workforce distribution, as other governmental physicians constitute 23% of emergency physicians, 19% of intensivists, and 22% of anesthesiologists nationwide. Regional data on the number of hospitals and inpatient beds across MOH and PS facilities were also extracted from the MOH Statistical Yearbook and used to calculate physician‐to‐bed ratios for each specialty and region. Physician‐to‐bed ratios served as a capacity‐based sensitivity analysis to complement population‐based density metrics and were not incorporated into inequality calculations. The dataset provides the most recent and comprehensive national statistics on physician workforce distribution, serving as a standardized foundation for evaluating sectoral and regional disparities across these three acute care disciplines.

## 3. Results

### 3.1. Physician Distribution by Specialty and Sector

Table 2 outlines the 2023 national distribution of emergency physicians, intensivists, and anesthesiologists across Saudi Arabia’s healthcare sectors. Emergency medicine had the largest number of physicians (4999), followed by anesthesia (4559) and intensive care (3185). Across all specialties, the MOH was the primary employer, accounting for 71% of intensivists, 65% of anesthesiologists, and 64% of emergency medicine physicians. The PS accounted for a smaller share of the workforce, employing only 13% of emergency physicians and 16% of intensivists, yet it accounted for 29% of anesthesia staffing. Other government sectors played a notable role in emergency care, accounting for 23% of that specialty’s workforce.

**TABLE 2:** Distribution of emergency physicians, intensivists, and anesthesiologists in Saudi Arabia by sector and gender, 2023.

Sector		Emergency physicians	Intensivists	Anesthesiologists
*MOH*				
	Male	2162 (67%)	1481 (72%)	1662 (74%)
	Female	1055 (33%)	565 (28%)	583 (26%)
	Total	**3217**	**2046**	**2245**

*PS*				
	Male	563 (86%)	479 (92%)	1088 (83%)
	Female	88 (14%)	41 (8%)	225 (17%)
	Total	**651**	**520**	**1313**

*Other governmental*				
	Male	900 (80%)	529 (85%)	855 (85%)
	Female	231 (20%)	90 (15%)	146 (15%)
	Total	**1131**	**619**	**1001**

*Overall*				
	Male	3625 (73%)	2489 (78%)	3605 (79%)
	Female	1374 (27%)	696 (22%)	954 (21
	Total	**4999**	**3185**	**4559**

*Note:* The bold values indicate the total number of physicians in each specialty within each sector, combining both male and female counts.

Abbreviations: MOH = Ministry of Health, PS = private sector.

This distribution underscores the MOH’s central role in staffing acute care services nationwide. Emergency medicine was primarily supported by the MOH, with 3217 physicians. In contrast, anesthesia had the largest share of PS physicians (1313), reflecting that sector’s focus on procedural and perioperative care. Intensive care staffing remained concentrated in the public sector, with only 16% (520 physicians) employed in the PS, highlighting limited PS participation in this highly specialized field.

When examining gender distribution, males constituted the majority across all specialties and sectors, accounting for approximately three‐quarters of the workforce, whereas female physicians accounted for roughly one‐quarter. Women’s representation was highest in emergency medicine (27%), followed by intensive care (22%) and anesthesia (21%). The MOH employed the largest number of female physicians, reflecting its role as the primary entry point for women into acute care fields. The PS showed comparatively higher female participation in anesthesia (17%), consistent with its procedural and perioperative focus, whereas women’s representation in intensive care within the PS remained limited.

### 3.2. Trends in the Physicians Workforce (2019–2023)

Between 2019 and 2023, Saudi Arabia demonstrated consistent growth in its acute care physician workforce across all three specialties, despite significant population increases during this period (Table 2). Emergency medicine showed the strongest expansion, with physician numbers increasing by 22.6% (from 4077 to 4999) and density rising from 13.6 to 14.8 per 100,000 residents. The number of anesthesiologists increased by 24.5% (from 3661 to 4559), with density rising from 12.2 to 13.5 per 100,000 population. Intensive care demonstrated the most significant growth rate, increasing by 24.8% (from 2552 to 3185 physicians). Although it remained the lowest‐density specialty among the three, it rose from 8.5 to 9.4 per 100,000 residents. Notably, the COVID‐19 pandemic period (2020–2021) coincided with sustained workforce expansion and national capacity‐building efforts.

### 3.3. Nationality Composition of the Workforce

Table 3 highlights notable variations in the proportion of Saudi nationals across both specialties and sectors. Emergency medicine had the highest concentration, with Saudis accounting for 46% of its workforce. Intensive care followed at 37%, while anesthesia recorded the lowest at just 22%. Within the MOH, nearly half of the emergency and intensivists were Saudi (49% and 47%, respectively), compared to only 25% in anesthesia. The PS had the lowest representation of Saudis across all specialties, particularly in intensive care (3%) and anesthesia (12%). These figures reflect an ongoing dependence on expatriate physicians, most pronounced in anesthesia and PS care.

**TABLE 3:** Nationality distribution of emergency physicians, intensivists, and anesthesiologists, Saudi Arabia, 2023.

Specialty	Overall	Saudis (total)	% of Saudis (total)	Saudis (MOH)	% of Saudis (MOH)	Saudis (PS)	% of Saudis (PS)	Saudis (other governmental)	% of Saudis (other governmental)
Emergency physicians	**4999**	**2277**	**46**	1569	49	201	31	507	45
Intensivists	**3185**	**1181**	**37**	960	47	16	3	205	33
Anesthesiologists	**4559**	**1005**	**22**	572	25	156	12	277	28

*Note:* Bold values indicate the overall total number of physicians, the total number of Saudi physicians, and the corresponding overall percentage of Saudis in each specialty across all sectors.

### 3.4. Regional Distribution and Density

Tables 4, 5, 6, and 7 present the distribution and density of physicians per 100,000 population across the Kingdom’s 20 health regions for the emergency, anesthesia, and intensive care specialties. In emergency medicine (Table 4), densities ranged from 22.8 per 100,000 in Al‐Jouf to 7.6 per 100,000 in Aseer, with a national average of 10.4 per 100,000. Riyadh and Jeddah had the highest total numbers of emergency physicians, 816 and 353, respectively, but lower per‐capita ratios of 9.1 and 8.5, reflecting urban population pressures. In contrast, less populated regions, such as Al‐Jouf, Qunfudah, and Al‐Baha, showed higher relative coverage despite having fewer physicians overall.

**TABLE 4:** Distribution and density of emergency physicians across regions in Saudi Arabia (MOH and PS).

Health region	MOH and PS	MOH	PS	Emergency physicians‐to‐100,000 people	Population
Al‐Jouf	96	95	1	**22.8**	420,597
Qunfudah	51	51	0	**18.0**	283,619
Al‐Bahah	61	61	0	**17.1**	355,922
Northern Borders	66	66	0	**16.8**	392,024
Qurayyat	33	33	0	**16.1**	204,645
Qaseem	201	188	13	**14.3**	1,402,158
Bishah	46	46	0	**14.2**	324,812
Medinah	309	282	27	**13.8**	2,243,555
Tabouk	126	121	5	**13.6**	929,788
Ha’il	105	100	5	**13.4**	783,263
Ta’if	136	126	10	**11.8**	1,155,966
Najran	72	70	2	**11.6**	621,547
Hafr Al‐Baten	54	52	2	**11.0**	490,067
Al‐Ahsa	116	62	54	**10.0**	1,158,795
Jazan	144	140	4	**9.8**	1,474,375
Eastern	345	235	110	**9.3**	3,729,473
The Holy Capital	259	235	24	**9.2**	2,810,039
Riyadh	816	557	259	**9.1**	9,016,005
Jeddah	353	232	121	**8.5**	4,167,942
Aseer	137	123	14	**7.6**	1,799,430
Overall	**3526**	**2875**	**651**	**10.4**	**33,764,022**

*Note:* Bold values indicate the overall total across all regions for the number of emergency physicians in MOH and PS sectors, the overall emergency physician density per 100,000 population, and the total population.

**TABLE 5:** Distribution of hospital beds and physician‐to‐bed ratios across regions in Saudi Arabia, 2023 (MOH and PS).

Health region	MOH beds	PS beds	Total MOH and PS beds	Emergency physicians to 100 beds	Intensivists to 100 beds	Anesthesiologists to 100 beds
Riyadh	9107	6345	15,452	**5.3**	**3.2**	**6.1**
Eastern	3456	3388	6844	**5.0**	**2.8**	**6.7**
Jeddah	3491	2830	6321	**5.6**	**4.7**	**6.9**
Medinah	3118	1025	4143	**7.5**	**5.8**	**4.9**
Qaseem	3324	363	3687	**5.5**	**3.7**	**4.7**
Aseer	2330	1170	3500	**3.9**	**3.7**	**4.4**
The Holy Capital	2694	703	3397	**7.6**	**8.2**	**5.8**
Jazan	2915	250	3165	**4.5**	**3.1**	**3.5**
Ta`if	2640	372	3012	**4.5**	**2.5**	**4.5**
Al ‐Ahsa	2055	930	2985	**3.9**	**3.4**	**5.4**
Ha`il	1940	256	2196	**4.8**	**2.0**	**4.3**
Tabouk	1920	135	2055	**6.1**	**4.1**	**4.3**
Najran	1300	250	1550	**4.6**	**3.3**	**4.4**
Northern Borders	1460	0	1460	**4.5**	**2.3**	**4.0**
Al‐Jouf	1330	30	1360	**7.1**	**3.8**	**4.3**
Al‐Bahah	1295	0	1295	**4.7**	**3.2**	**4.6**
Hafr Al‐Baten	1000	150	1150	**4.7**	**2.3**	**3.7**
Bishah	920	0	920	**5.0**	**4.1**	**3.9**
Qurayyat	610	0	610	**5.4**	**1.8**	**2.8**
Qunfudah	400	0	400	**12.8**	**5.5**	**6.8**
Overall	**9107**	**18,197**	**27,304**	**5.7**	**3.7**	**4.8**

*Note:* Bold values in the Overall row indicate the total number of beds across all regions, while bold values in the physician‐to‐bed columns indicate the specialty‐specific physician‐to‐100‐bed ratios for each region and overall.

**TABLE 6:** Distribution and density of intensivists across regions in Saudi Arabia (MOH and PS).

Health region	MOH and PS	MOH	PS	Intensivists‐to‐100,000 people	Population
Al‐Jouf	51	50	1	**12.1**	420,597
Al‐Bahah	42	42	0	**11.8**	355,922
Bishah	38	38	0	**11.7**	324,812
Medinah	242	189	53	**10.8**	2,243,555
The Holy Capital	279	259	20	**9.9**	2,810,039
Qaseem	138	120	18	**9.8**	1,402,158
Tabouk	84	81	3	**9.0**	929,788
Al‐Ahsa	102	72	30	**8.8**	1,158,795
Northern Borders	34	34	0	**8.7**	392,024
Najran	51	45	6	**8.2**	621,547
Qunfudah	22	22	0	**7.8**	283,619
Aseer	131	101	30	**7.3**	1,799,430
Jeddah	299	175	124	**7.2**	4,167,942
Jazan	97	82	15	**6.6**	1,474,375
Ta’if	76	68	8	**6.6**	1,155,966
Ha’il	45	32	13	**5.7**	783,263
Riyadh	494	353	141	**5.5**	9,016,005
Qurayyat	11	11	0	**5.4**	204,645
Hafr Al‐Baten	26	24	2	**5.3**	490,067
Eastern	194	138	56	**5.2**	3,729,473
Overall	**2456**	**1936**	**520**	**7.3**	**33,764,022**

*Note:* Bold values indicate the overall total across all regions for the number of intensivists in MOH and PS sectors, the overall intensivist density per 100,000 population, and the total population.

**TABLE 7:** Distribution and density of anesthesiologists across regions in Saudi Arabia (MOH and PS).

Health region	MOH and PS	MOH	PS	Anesthesiologists‐to‐100,000 people	Population
Al‐Bahah	60	59	1	**16.9**	355,922
Northern Borders	59	59	0	**15.1**	392,024
Al‐Ahsa	160	113	47	**13.8**	1,158,795
Al‐Jouf	58	57	1	**13.8**	420,597
Qaseem	173	136	37	**12.3**	1,402,158
Eastern	456	231	225	**12.2**	3,729,473
Ha’il	94	78	16	**12.0**	783,263
Ta’if	136	118	18	**11.8**	1,155,966
Bishah	36	34	2	**11.1**	324,812
Najran	68	58	10	**10.9**	621,547
Riyadh	944	450	494	**10.5**	9,016,005
Jeddah	439	162	277	**10.5**	4,167,942
Tabouk	88	79	9	**9.5**	929,788
Qunfudah	27	27	0	**9.5**	283,619
Medinah	202	141	61	**9.0**	2,243,555
Hafr Al‐Baten	43	35	8	**8.8**	490,067
Aseer	155	98	57	**8.6**	1,799,430
Qurayyat	17	17	0	**8.3**	204,645
Jazan	111	96	15	**7.5**	1,474,375
The Holy Capital	198	163	35	**7.0**	2,810,039
Overall	**3524**	**2211**	**1313**	**10.4**	**33,764,022**

*Note:* Bold values indicate the overall total across all regions for the number of anesthesiologists in MOH and PS sectors, the overall anesthesiologist density per 100,000 population, and the total population.

When physician distribution is examined relative to hospital bed capacity rather than population size (Table 5), regional patterns reveal the relationship between workforce deployment and healthcare infrastructure. Nationally, there were 5.7 emergency physicians, 3.7 intensivists, and 4.8 anesthesiologists per 100 beds across MOH and PS facilities combined. Emergency physician‐to‐bed ratios ranged from 12.8 per 100 beds in Qunfudah to 3.9 per 100 beds in Al‐Ahsa and Aseer, with substantial regional variation. Intensivist‐to‐bed ratios were lowest in Qurayyat (1.8 per 100 beds) and Ha’il (2.0 per 100 beds), while the Holy Capital demonstrated the highest ratio at 8.2 per 100 beds. Anesthesiologist‐to‐bed ratios ranged from 2.8 per 100 beds in Qurayyat to 6.9 per 100 beds in Jeddah, with major urban regions including Riyadh (6.1), Eastern (6.7), and Jeddah (6.9) maintaining higher staffing ratios than their bed capacities. Notably, regions with high population‐based densities, such as Qunfudah and Al‐Jouf, demonstrated correspondingly elevated bed‐based ratios for emergency medicine (12.8 and 7.1 per 100 beds, respectively), reflecting limited bed capacity in these peripheral areas. In contrast, large urban centers such as Riyadh and the Eastern Region exhibited moderate bed‐based ratios despite employing the largest absolute numbers of physicians, underscoring the substantial hospital infrastructure in these regions.

According to Table 6, regional intensivists’ density ranged widely, with a population‐weighted national average of 9.4 per 100,000 when all sectors were combined (Table 2). Al‐Jouf (12.1), Al‐Bahah (11.8), and Bishah (11.7) recorded the highest relative coverage. In contrast, major cities such as Riyadh (5.5), Jeddah (7.2), and the Eastern Region (5.2) had lower ratios despite their large populations. PS involvement in intensive care was most notable in Riyadh (*n* = 141), Jeddah (*n* = 124), and the Eastern Region (*n* = 56). In contrast, several other regions had no intensivists employed in the PS.

As in emergency medicine, the regional variation in the distribution of intensivists must be interpreted in light of hospital infrastructure. Peripheral regions demonstrate higher per‐capita ratios due to smaller service networks, while large urban regions support extensive acute care infrastructure with comparatively lower population‐adjusted physician densities.

The distribution of anesthesiologists, as shown in Table 7, mirrored patterns observed in emergency care, with the highest densities in Al‐Bahah (16.9), the Northern Borders (15.1), and Al‐Ahsa (13.8). The national average stood at 10.4 per 100,000 population. While Riyadh and Jeddah had the largest absolute numbers of anesthesiologists, their density remained moderate at 10.5 per 100,000 population. Staffing in the PS was heavily concentrated in urban areas, contributing 494 physicians in Riyadh and 277 in Jeddah.

When viewed alongside Table 5, the density of anesthesiologists appears more closely related to hospital and surgical capacity than population size alone. Smaller regions with limited hospital infrastructure, such as Al‐Bahah and the Northern Borders, show higher per‐capita ratios despite modest staffing levels. In contrast, major urban regions maintain moderate densities despite employing the largest absolute numbers of anesthesiologists.

### 3.5. Inequality Analysis: Lorenz Curves and Gini Coefficients

**FIGURE 1:**
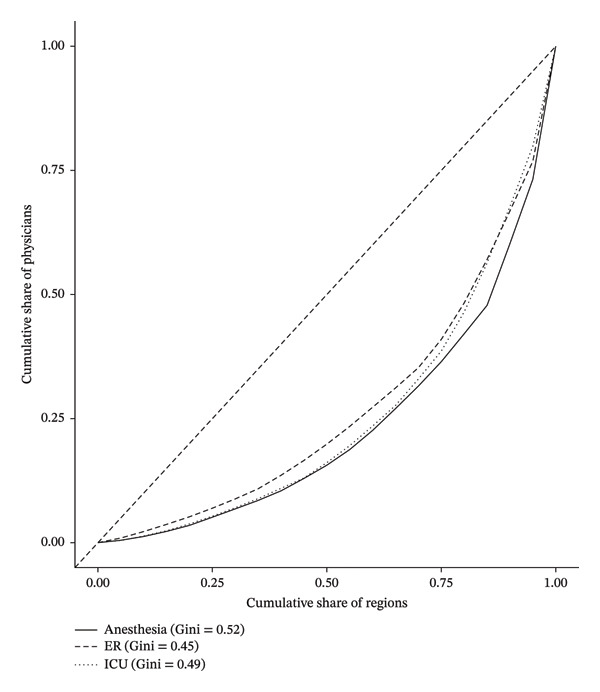
Lorenz curves comparing emergency physicians, intensivists, and anesthesiologists by region, 2023.

Figure 1 presents Lorenz curves comparing the regional distributions of emergency physicians, intensivists, and anesthesiologists across Saudi Arabia in 2023. The analysis reveals a consistent pattern of moderate inequality across all three specialties, with Gini coefficients of 0.45 for emergency medicine, 0.49 for intensive care, and 0.52 for anesthesia. While the differences are relatively small, anesthesiologists were slightly more unevenly distributed, followed closely by physicians in intensive care and emergency medicine. These findings highlight systemic imbalances in the regional availability of acute care specialties and underscore the need for more equitable workforce planning across the Kingdom’s health regions.

**FIGURE 2:**
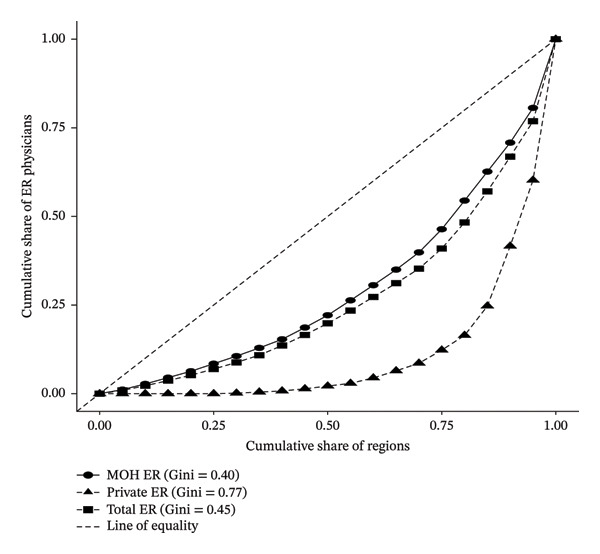
Lorenz curve showing the geographic distribution of emergency physicians in Saudi Arabia by sector, 2023.

**FIGURE 3:**
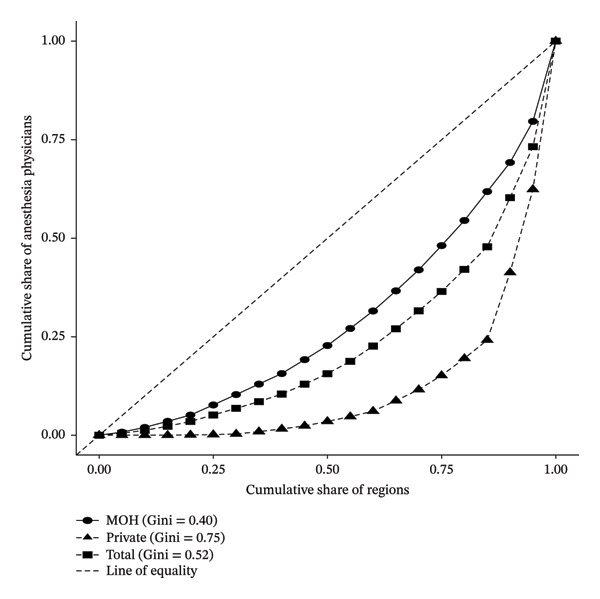
Lorenz curve showing the geographic distribution of anesthesiologists in Saudi Arabia by sector, 2023.

**FIGURE 4:**
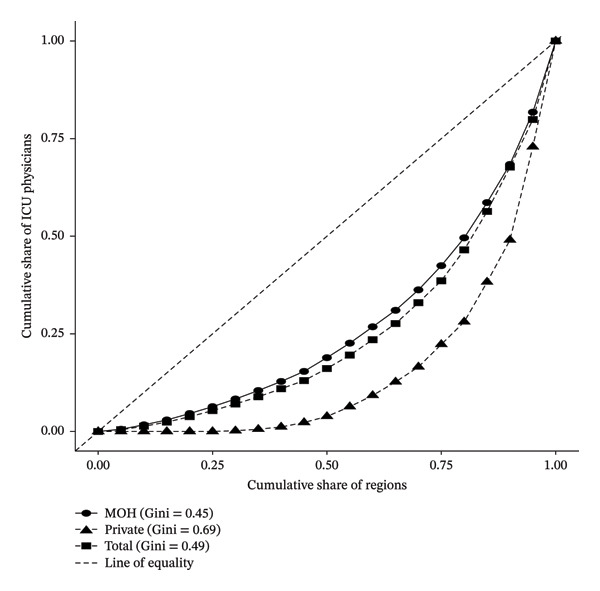
Lorenz curve showing the geographic distribution of intensivists in Saudi Arabia by sector, 2023.

Figures 2, 3, and 4 illustrate the Lorenz curves of physician distribution across emergency medicine, anesthesia, and intensive care, disaggregated by sector. In emergency medicine (Figure 2), the overall Gini coefficient was 0.45, indicating moderate geographic inequality. MOH‐employed emergency physicians were more evenly distributed, with a Gini of 0.40. In contrast, PS physicians in this specialty were highly concentrated in select urban regions, as reflected in a Gini coefficient of 0.77.

Anesthesia, as shown in Figure 3, exhibited the most significant inequality among the three fields, with an overall Gini index of 0.52. MOH anesthetists were moderately distributed (Gini = 0.40), while the PS demonstrated significant concentration in metropolitan areas, as indicated by a Gini coefficient of 0.75.

Intensivists (Figure 4) had an overall Gini of 0.49. Similar to the other specialties, MOH distribution was more balanced (Gini coefficient = 0.45), whereas PS distribution remained uneven (Gini coefficient = 0.69), with services concentrated in major cities.

Collectively, the Lorenz curve and Gini coefficient analyses reveal a consistent pattern of geographic inequality in physician distribution across all three specialties, with the PS exhibiting the highest levels of concentration in urban centers. While the MOH has maintained comparatively balanced deployment, significant disparities persist, particularly in anesthesia and intensive care. These findings highlight the structural imbalance in workforce allocation and underscore the need for targeted policies to promote more equitable access to intensive care services across all regions and sectors.

## 4. Discussion

### 4.1. Key Findings

This national analysis of Saudi Arabia’s 2023 physician workforce in emergency medicine, anesthesia, and intensive care demonstrates that the Kingdom has achieved favorable national physician densities exceeding international benchmarks. Yet, significant disparities persist in geographic distribution, sectoral balance, and workforce localization. The study identified 4999 emergency medicine physicians, 4559 anesthesiologists, and 3185 intensivists, with approximately 65% employed by the MOH and minimal PS participation in intensive care (16%). Male physicians predominated across all specialties (73%–79%), while Saudization rates varied substantially: 46% in emergency medicine, 37% in intensive care, and only 22% in anesthesia. The PS showed remarkably low localization rates, with 3% in intensive care, 12% in anesthesia, and 31% in emergency medicine. These localization patterns mirror the sectoral dependence observed in Table 3, in which the MOH demonstrates substantially higher Saudization rates than the PS, particularly in intensive care and anesthesia. This imbalance suggests that workforce localization remains closely tied to public‐sector employment, with limited penetration into private and specialized care settings. Taken together, these findings confirm that Saudi Arabia has achieved favorable national physician densities in acute care specialties, while inequities persist primarily at the geographic and sectoral levels.

Geographic distribution revealed significant inequality, with Gini coefficients of 0.52 for anesthesia, 0.49 for intensive care, and 0.45 for emergency medicine. While urban centers such as Riyadh and Jeddah had the highest absolute numbers of physicians, their per‐capita densities were lower than in peripheral regions such as Al‐Jouf and Al‐Bahah. However, these smaller regions lacked clinical specialization and sectoral balance, with PS services heavily concentrated in major cities. These disparities were most pronounced in the PS across all three specialties. Importantly, the apparent regional differences in physician density should be interpreted in light of hospital and bed distribution, as peripheral regions with fewer hospitals and beds may exhibit higher population‐adjusted physician ratios despite limited absolute service capacity. These findings indicate that workforce expansion has been successful at the national level and that inequities are primarily driven by geographic maldistribution, sectoral concentration, and localization gaps rather than absolute shortages. This distinction is critical for policy formulation: Saudi Arabia does not need to increase the total number of acute care physicians substantially, but rather to redistribute existing and future workforce capacity more equitably across regions and sectors.

### 4.2. Workforce Distribution and Capacity Alignment Challenges

Although Saudi Arabia’s national physician densities in emergency medicine, anesthesia, and intensive care exceed commonly cited global benchmarks, challenges persist in aligning workforce distribution with service demand, particularly in intensive care and anesthesia. As the population ages and demand for long‐term and acute care rises [5], the concentration of intensivists within specific regions and sectors increasingly challenges alignment between workforce deployment and regional service demand. These gaps are further stressed by demographic shifts and the concentration of patients in urban areas, which increase service demand without matching workforce growth [21]. Major hospitals, particularly in cities, face additional pressure from long‐stay patients occupying beds that could be used for acute cases [5]. Compounding the issue, the underdevelopment of primary healthcare in urban centers such as Jeddah often redirects patients to overburdened tertiary hospitals [25], thereby intensifying strain on already stretched intensive care and anesthesia teams. This mismatch between physician supply and service demand is further accentuated by the concentration of hospital beds and tertiary facilities in major urban regions, where workforce growth has not kept pace with expanding infrastructure and patient volumes.

### 4.3. Inequitable Distribution and Regional Disparities

Despite ongoing reform initiatives, the geographic distribution of physicians in Saudi Arabia remains uneven. Regions such as Al‐Jouf, Al‐Bahah, and Qurayyat report higher physician‐to‐population ratios than major cities such as Riyadh and Jeddah, not due to overstaffing but rather to their smaller populations. In contrast, urban centers face mounting pressure on their healthcare systems, where large absolute numbers of physicians mask lower per‐capita coverage. Gini coefficients further illustrate this imbalance, particularly in the PS, where anesthesia services are disproportionately concentrated in Riyadh and Jeddah [26]. This urban reliance contributes to delays in accessing timely care, especially for emergency and critical interventions. During the COVID‐19 pandemic, intensive care e‐referral data highlighted persistent regional disparities in demand, confirming that many peripheral regions remain under‐resourced despite seemingly favorable coverage ratios [27]. To address these gaps, workforce planning must move beyond simple headcounts and instead focus on population‐adjusted needs and regional service demands [4]. As shown in Table 5, large urban regions host the majority of hospitals and inpatient beds nationwide, underscoring that lower physician‐to‐population ratios in these areas coexist with substantially higher service complexity and institutional workload. This pattern indicates that higher physician‐to‐population ratios in peripheral regions reflect smaller population denominators rather than greater service capacity. In contrast, urban regions are subject to sustained pressure from higher patient volumes and greater infrastructure concentration. This reinforces the need to interpret physician‐to‐population ratios alongside the distribution of infrastructure to avoid overstating service availability in low‐capacity regions.

PS involvement in emergency medicine and intensive care remains minimal, with only 13% of emergency physicians and 16% of intensivists working in this sector. Several barriers likely contribute to this gap. Intensive care units require significant capital investment and ongoing operational costs, which can discourage private hospitals, where profitability is often linked to elective and procedural services [28]. Additionally, the lack of targeted incentives or reimbursement mechanisms for PS providers reduces their willingness to expand into emergency and intensive care, where profit margins are generally lower [29]. Regulatory requirements and reliance on public‐sector subsidies for emergency and ICU services further reinforce this imbalance [30]. Consequently, patients in regions underserved by the MOH face limited access to timely acute care when PS providers are unable or unwilling to supply these services. Overcoming these barriers through financial incentives, public–private partnerships, and policy reforms is crucial to fostering greater PS involvement in high‐acuity specialties, ultimately improving access across the Kingdom. This structural imbalance reinforces regional dependence on the MOH, as PS facilities remain limited in number and in bed capacity outside major metropolitan areas.

### 4.4. Saudization, Training, and Retention Barriers

Efforts to increase Saudization within the physician workforce have yielded mixed results, despite overall workforce expansion. As demonstrated in Table 3, Saudization is consistently higher within the MOH across all specialties, whereas the PS exhibits persistently low localization, particularly in intensive care and anesthesia. Emergency medicine has the highest national localization rate at 46%, whereas anesthesia and intensive care have lower rates of 22% and 37%, respectively. These trends reflect findings by Alnowibet et al. [31], who reported that expatriates continue to dominate the PS, particularly in specialized roles. Cultural and lifestyle factors also influence career choices, with many anesthesia residents showing limited interest in domestic or academic roles and favoring international opportunities instead [19]. Structured training programs and retention strategies remain underdeveloped, further limiting progress. Addressing these gaps will require comprehensive reforms, including enhancing training pipelines, incentivizing rural placements, and increasing public awareness, particularly for less visible specialties such as anesthesia [32]. Strengthening academic and professional pathways is essential to meeting the Vision 2030 workforce targets [33].

### 4.5. Policy and System‐Level Considerations

Saudi Arabia’s ongoing healthcare transformation must also address broader structural reforms. The Model of Care under Vision 2030 promotes alignment between service delivery and population health needs across six defined care systems [4, 34]. Yet implementation remains uneven, particularly in physician distribution. Public feedback highlights persistent concerns about responsiveness and engagement in emergency departments, indicating systemic inefficiencies despite policy efforts [11]. While digital tools and AI offer significant potential, their adoption has been limited; 61.47% of anesthesiologists report that no formal AI strategies are in place at their institutions [35]. International examples also illustrate the risks of mismatched workforce planning, as seen in U.S. projections of a surplus of emergency physicians by 2030 [36]. To avoid similar imbalances, Saudi Arabia must continue refining its workforce strategies by using accurate, data‐driven demand forecasts to close current gaps and anticipate future needs. Aligning workforce deployment with hospital and bed expansion and simultaneously addressing sectoral Saudization gaps will be critical to achieving an equitable and sustainable workforce distribution under Vision 2030.

### 4.6. Implications for Emergency Medicine System Performance

The geographic maldistribution documented in this study has direct implications for emergency care delivery and system resilience. Regions with the lowest emergency physician densities, Aseer (7.6 per 100,000), Jeddah (8.5 per 100,000), and Riyadh (9.1 per 100,000), are major urban centers experiencing the highest patient volumes. This workforce‐demand mismatch risks compromising time‐sensitive emergency interventions and exacerbating challenges in ED responsiveness [11]. The 23% concentration of emergency physicians in other governmental facilities, excluded from the regional analysis due to a lack of geographic attribution, represents an essential gap in assessing actual emergency response capacity. Furthermore, the marked private‐sector concentration in major cities (Gini coefficient of 0.77) constrains regional resilience, as peripheral areas lack alternative care pathways when MOH facilities face surge demand. Optimal emergency physician deployment, informed by both population‐based and capacity‐based metrics, is essential to ensuring equitable access to time‐sensitive emergency care and to strengthening system preparedness aligned with Vision 2030 objective [12].

### 4.7. Study Limitations

This study has several limitations that should be acknowledged. First, the analysis relies primarily on secondary data obtained from the MOH Statistical Yearbook, which may not fully capture physician workforce dynamics across all governmental sectors. Although physicians from other governmental entities were included in national totals, their exclusion from regional analyses because of a lack of geographic attribution may underestimate workforce availability in certain regions. Second, using a single primary data source limits the ability to validate findings against alternative datasets, such as those from the Saudi Commission for Health Specialties or other governmental healthcare systems. Third, the cross‐sectional design, based on a single year of data, precludes assessing temporal trends or drawing causal inferences about workforce changes over time. Fourth, the absence of demand‐side indicators, such as emergency department visit volumes, ICU bed capacity, or surgical caseloads, limits the ability to align physician distribution with actual service utilization and population health needs.

Additionally, using a population‐based denominator may not fully capture specialty‐specific service demand for anesthesia and intensive care, which is more directly influenced by surgical activity, ICU capacity, and hospital case mix. Fifth, the inequality analysis employed non‐population‐weighted data, measuring geographic concentration across administrative regions rather than per‐capita accessibility. While this quantifies workforce maldistribution across boundaries, population‐weighted measures would better reflect patient‐centered equity of access. These limitations highlight the need for integrated, multisource workforce datasets and longitudinal analyses to support more precise workforce planning and policy development.

## 5. Conclusion

This study demonstrates that, despite measurable growth and favorable national physician densities in emergency medicine, intensive care, and anesthesia between 2019 and 2023, persistent disparities in geographic distribution, sectoral balance, and localization remain across Saudi Arabia. The MOH remains the dominant employer of acute care physicians, while PS participation, particularly in intensive care, remains limited. Urban regions host the largest absolute numbers of physicians and the majority of hospitals and inpatient beds, yet continue to exhibit lower population‐adjusted physician densities due to high service demand and infrastructure concentration. In contrast, peripheral regions exhibit higher per‐capita physician ratios but lack clinical specialization, private‐sector presence, and institutional capacity. These findings reaffirm that equitable access to acute care in Saudi Arabia depends less on increasing the number of physicians and more on optimizing geographic distribution, sectoral participation, and localization strategies.

Geographic inequality, as reflected by moderate Gini coefficients, remains most pronounced in anesthesia and intensive care, particularly within the PS. Saudization progress has been uneven, with continued reliance on expatriate physicians in anesthesia and intensive care, especially outside the public sector. These findings indicate that workforce expansion alone is insufficient to address inequity without parallel alignment of training pipelines, sectoral incentives, and infrastructure planning. As population growth and aging continue to increase demand for high‐acuity services, data‐driven workforce policies that integrate physician supply, hospital capacity, and localization strategies will be essential to achieving a more equitable, resilient, and sustainable acute care workforce, aligned with Vision 2030 and the objectives of the NTP.

## Author Contributions

Waleed M. Kattan was solely responsible for all aspects of this research, including study conceptualization, methodology design, data collection and analysis, interpretation of results, manuscript preparation, and critical revision of intellectual content.

## Funding

This research received no external funding. The study was conducted independently without financial support from governmental, commercial, or nonprofit organizations. The APC was funded by the Deanship of Scientific Research (DSR) at King Abdulaziz University. The authors, therefore, acknowledge with thanks the technical and financial support.

## Ethics Statement

Before commencing the study, administrative permissions were obtained to ensure appropriate access to national administrative data. As this study used publicly available, de‐identified, aggregate data from the Ministry of Health’s statistical Yearbook 2023, formal ethical approval was not required under national research regulations for secondary analysis of anonymized national statistics. Ethical considerations were carefully addressed to ensure data confidentiality, secure handling, and compliance with institutional and national research governance regulations. No individual‐level data or personally identifiable information was accessed or analyzed in this study.

## Conflicts of Interest

The author declares no conflicts of interest.

## Data Availability

The data supporting the findings of this study are publicly available from the Ministry of Health of the Kingdom of Saudi Arabia. The primary data source, the Saudi MOH Statistical Yearbook 2023, is available at https://www.moh.gov.sa/en/Ministry/Statistics/book/Pages/default.aspx. All data used in this study are derived from publicly accessible national statistics. Aggregated datasets and analytical code used for calculating physician densities, Lorenz curves, and Gini coefficients are available from the corresponding author upon reasonable request.
